# DNA barcode analysis of butterfly species from Pakistan points towards regional endemism

**DOI:** 10.1111/1755-0998.12131

**Published:** 2013-06-24

**Authors:** Muhammad Ashfaq, Saleem Akhtar, Arif M Khan, Sarah J Adamowicz, Paul D N Hebert

**Affiliations:** * Biodiversity Institute of Ontario, University of GuelphGuelph, Ontario, Canada, N1G 2W1; † National Institute for Biotechnology and Genetic EngineeringJhang Road, Faisalabad, Pakistan

**Keywords:** COI, endemism, Lepidoptera, mtDNA, Pakistan

## Abstract

DNA barcodes were obtained for 81 butterfly species belonging to 52 genera from sites in north-central Pakistan to test the utility of barcoding for their identification and to gain a better understanding of regional barcode variation. These species represent 25% of the butterfly fauna of Pakistan and belong to five families, although the Nymphalidae were dominant, comprising 38% of the total specimens. Barcode analysis showed that maximum conspecific divergence was 1.6%, while there was 1.7–14.3% divergence from the nearest neighbour species. Barcode records for 55 species showed <2% sequence divergence to records in the Barcode of Life Data Systems (BOLD), but only 26 of these cases involved specimens from neighbouring India and Central Asia. Analysis revealed that most species showed little incremental sequence variation when specimens from other regions were considered, but a threefold increase was noted in a few cases. There was a clear gap between maximum intraspecific and minimum nearest neighbour distance for all 81 species. Neighbour-joining cluster analysis showed that members of each species formed a monophyletic cluster with strong bootstrap support. The barcode results revealed two provisional species that could not be clearly linked to known taxa, while 24 other species gained their first coverage. Future work should extend the barcode reference library to include all butterfly species from Pakistan as well as neighbouring countries to gain a better understanding of regional variation in barcode sequences in this topographically and climatically complex region.

## Introduction

DNA barcoding has emerged as a useful tool for the identification and discovery of animal species. It employs sequence diversity in a 648 base pair fragment near the 5′ end of the mitochondrial cytochrome *c* oxidase subunit I (COI) gene as a tool for species discrimination (Hebert *et al*. 2003a). Barcoding has been shown to discriminate species across the animal kingdom (Tyagi *et al*. 2010; Virgilio *et al*. 2010) including fishes, mammals, birds, insects, crustaceans and many other groups (Hebert *et al*. 2004a; Foottit *et al*. 2008; Hastings *et al*. 2008; Hubert *et al*. 2008; Hou *et al*. 2009; Wong *et al*. 2009; Clare *et al*. 2011). Reflecting the rapid growth in barcode coverage (Jinbo *et al*. 2011), BOLD, the Barcode of Life Data System (Ratnasingham & Hebert 2007), now includes records for more than 261K animal species. The order Lepidoptera has received particular attention (Hajibabaei *et al*. 2006; Silva-Brandao *et al*. 2009; Hebert *et al*. 2010; Kim *et al*. 2010) with 691K barcode records on BOLD (Feb 3, 2013), including data for 9124 named butterfly (Papilionoidea, Hesperioidea) species from 194 countries.

The gap between maximum intraspecific and minimum interspecific distances has been used for species delimitation in various animal groups (Hebert *et al*. 2004a; Meyer & Paulay 2005; Meier *et al*. 2006, 2008; Puillandre *et al*. 2012). This approach has helped to resolve cryptic species complexes (Hebert *et al*. 2004b; Burns *et al*. 2007; Park *et al*. 2011; Deng *et al*. 2012) and has aided ecological studies (Valentini *et al*. 2009; Pramual & Kuvangkadilok 2012). For example, Vaglia *et al*. (2008) used DNA barcodes to reveal cryptic species of sphingid moths, while van Nieukerken *et al*. (2012) discriminated cryptic species of leaf-mining Lepidoptera. Likewise, Carletto *et al*. (2009) discriminated sibling species of *Aphis gossypii*.

The effectiveness of DNA barcoding has spurred efforts to construct DNA barcode reference libraries for various animal groups (Ekrem *et al*. 2007; Guralnick & Hill 2009; Janzen *et al*. 2009; Lee *et al*. 2011; Zhou *et al*. 2011; Webb *et al*. 2012). These libraries not only aid the documentation of biodiversity (Janzen *et al*. 2005; Naro-Maciel *et al*. 2010) including endangered species (Elmeer *et al*. 2012; Vanhaecke *et al*. 2012), but can disclose endemism (Bossuyt *et al*. 2004; Quilang *et al*. 2011; Sourakov & Zakharov 2011). Because Lepidoptera have been selected as a model group for intensive analysis, the order is well represented on BOLD, but some regions such as South-East Asia have seen little investigation. Barcode records are available for a significant fraction of the Central Asian butterfly fauna (Lukhtanov *et al*. 2009) and for a smaller number of species from Western India (Gaikwad *et al*. 2012). However, these studies fail to provide coverage for many species known from Pakistan (Roberts 2001). The current study had the primary goals of testing the effectiveness of DNA barcodes in the identification of butterfly species from Pakistan and comparing these records with those from other regions to gain a better sense of the extent of intraspecific variation.

## Materials and methods

### Specimen sampling

**Fig 1:**
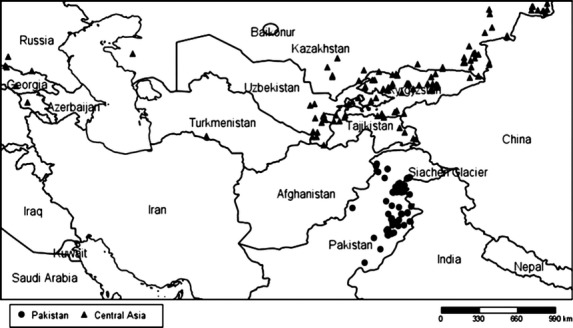
Map of Pakistan and neighbouring nations showing collection localities for this study as well those for specimens examined in a prior study (Lukhtanov *et al*. 2009).

Butterflies were collected at 107 locations across central and northern Pakistan (Fig. 1) during 2009–2012. These sites included three different climatic zones: tropical, subtropical and temperate, with altitudes ranging from 127 to 2660 m, and both agricultural and forested environments. Each specimen was labelled, assigned a code number and deposited in the arthropod collection at the National Institute for Biotechnology and Genetic Engineering (NIBGE), Faisalabad, for subsequent morphological and molecular analysis. Using standard guides to the fauna (Malik 1973; Hasan 1994; Roberts 2001), the 407 specimens were assigned to 81 species belonging to 52 genera. Two species (*Lasiommata* sp. MA01 and *Polycaena* sp. MA01) could only be identified to a generic level, but were included in the analysis. Specimen data and images are available on BOLD (Ratnasingham & Hebert 2007) in the project MABUT (Barcoding Butterflies of Pakistan). Fifty-nine of the 81 species were represented by more than one specimen (range 2–20). All sequences generated in this study are available on BOLD (Process IDs: MABUT001-10 to MABUT312-12; MABUT326-13 to MABUT388; MAIMB133-09 to MAIMB137-09, 166-09, 167-09, 169-09, 170-09, 178-09, 179-09) and on GenBank under the following accession nos: KC158311–KC158471, HQ990321–HQ990449, HQ990705, HQ990728–HQ990729, GU681850–GU681851, GU681855–GU681856, GU681859, GU681870 and GU681872–GU681875.

### DNA extractions and PCR amplifications

A single leg was removed from each specimen with a sterile forceps and transferred to a 96-well microplate preloaded with 30 μL of 95% ethanol in each well. DNA extraction, PCR amplification and sequencing were performed at the Canadian Centre for DNA Barcoding (CCDB) following standard protocols (Ivanova *et al*. 2006, 2007; Ivanova & Grainger 2007a,b2007b,c2007c). DNA extractions were performed by following the protocols developed for invertebrate barcoding (Ivanova *et al*. 2006). Amplification of the COI-5′ barcode region was performed with primer pair LepF1 (ATTCAACCAATCATAAAGATATTGG)/LepR1 (TAAACTTCTGGATGTCCAAAAAATCA) (Hebert *et al*. 2004b) using the following PCR conditions: 94 °C (1 min); 5 cycles of 94 °C (30 s), 45 °C (40 s), 72 °C (1 min); 35 cycles of 94 °C (30 s), 51 °C (40 s), 72 °C (1 min); and final extension of 72 °C (10 min). PCRs were carried out in 12.5 μL reactions containing standard PCR ingredients and 2 μL of DNA template. PCR products were analysed on 2% agarose E-gel® 96 system (Invitrogen Inc.). Amplicons were sequenced bidirectionally using BigDye Terminator Cycle Sequencing Kit (v3.1) on an ABI 3730XL DNA Analyzer. The forward and the reverse sequences were assembled and aligned using CodonCode Aligner (CodonCode Corporation, USA). Sequences were also inspected and translated in mega V5 (Tamura *et al*. 2011) to verify that they were free of stop codons and gaps.

### Data analysis

The sequence from each specimen was compared with barcode sequences on GenBank using ‘Blast’ and with sequences on BOLD using the ‘Identification Request’ function. Prior studies have revealed that most different species of Lepidoptera show >2% sequence divergence at CO1 (Hebert *et al*. 2003b), and researchers have used a 2% pairwise distance threshold for species delimitation (Strutzenberger *et al*. 2011). For the barcode-based identity analysis, we also used a threshold of 2% divergence. DNA barcodes for 9124 butterfly species from 194 countries are currently available on BOLD, all readily available for sequence comparisons. In addition, the results were compared with those of prior studies in Central Asia (353 butterfly species) (Lukhtanov *et al*. 2009), Korea (83 species) (Kim *et al*. 2010) and India (40 species) (Gaikwad *et al*. 2012). ClustalW nucleotide sequence alignments (Thompson *et al*. 1994) and NJ clustering analysis were performed using mega V5 (Tamura *et al*. 2011). The Kimura-2-Parameter (K2P) (Kimura 1980) distance model was used, along with pairwise deletion of missing sites, with nodal support estimated using 500 bootstrap replicates. The online version of Automatic Barcode Gap Discovery (ABGD) (Puillandre *et al*. 2012) was used for both pairwise distance analyses and to generate distance histograms and distance ranks. The presence or absence of a ‘barcode gap’ (Meyer & Paulay 2005) was also determined for each species as a test of the reliability of its discrimination. Using the barcode gap criterion, a species is distinct from its nearest neighbour (NN) if its maximum intraspecific distance is less than the distance to its NN sequence. The ‘Barcode Gap Analysis’ (BGA) was performed using BOLD. Species identification success by ‘Best Match’ and cluster analysis was performed using TaxonDNA (Meier *et al*. 2006). The relationship between geographical distance and intraspecific genetic distance was analysed separately for each species (with at least three individuals and three locations) using the Mantel test (Mantel 1967) and by linear regression using xlstat (version 2013.3.02; Addinsoft, Inc., NY, USA).

## Results

Barcode sequences greater than 500 base pairs (bp) were recovered from 374 of the 407 specimens (92%), providing at least one sequence for each of the 81 butterfly species. When these sequences were compared with those in the BOLD and NCBI databases, close sequence matches (<2% divergence) were detected for 55 of the species from Pakistan, while 26 lacked a match. The highest number of matches involved records from India (15), Central Asia (11) and Korea (10).

**Fig 2:**
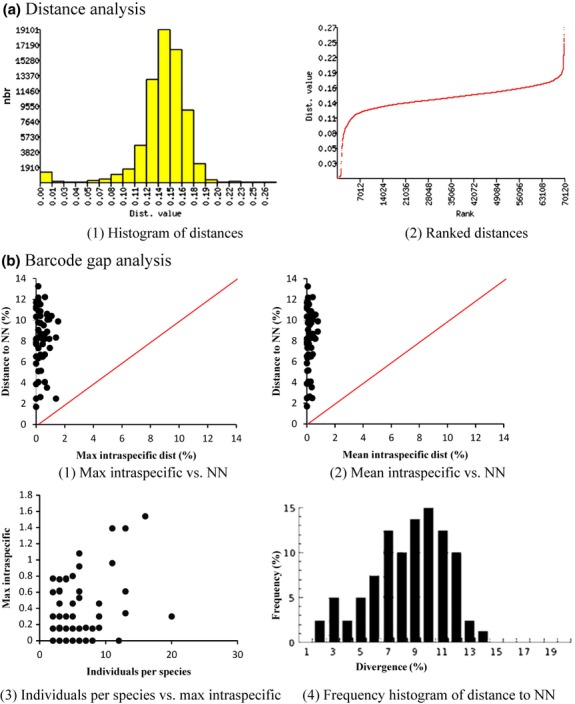
Pairwise distance divergence (%) (a) and barcode gap analysis (b) for butterflies from Pakistan as generated by Automatic Barcode Gap Discovery (Puillandre *et al*. 2012) and by BOLD (Ratnasingham & Hebert 2007), respectively. NN = nearest neighbour.

Figure 2 presents results from the ABGD and BGA analyses. Distance values show a gap between the intraspecific and the interspecific distances (Fig. 2A). As well, both the maximum and mean distances to NN are higher than the respective intraspecific distances for all species (Fig. 2B). Nearest neighbour distances were more than 3% for all but three species pairs: *Tarucus balkanicus* vs. *T. rosaceus* (1.70%), *Junonia orithya* vs. *J. hierta* (2.49%) and *Celastrina huegelii* vs. *C. argiolus* (2.64%). Intraspecific distances could not be determined for the 22 species with just a single representative, but NN distances were greater than 4% for 21 of them.

**Fig 3:**
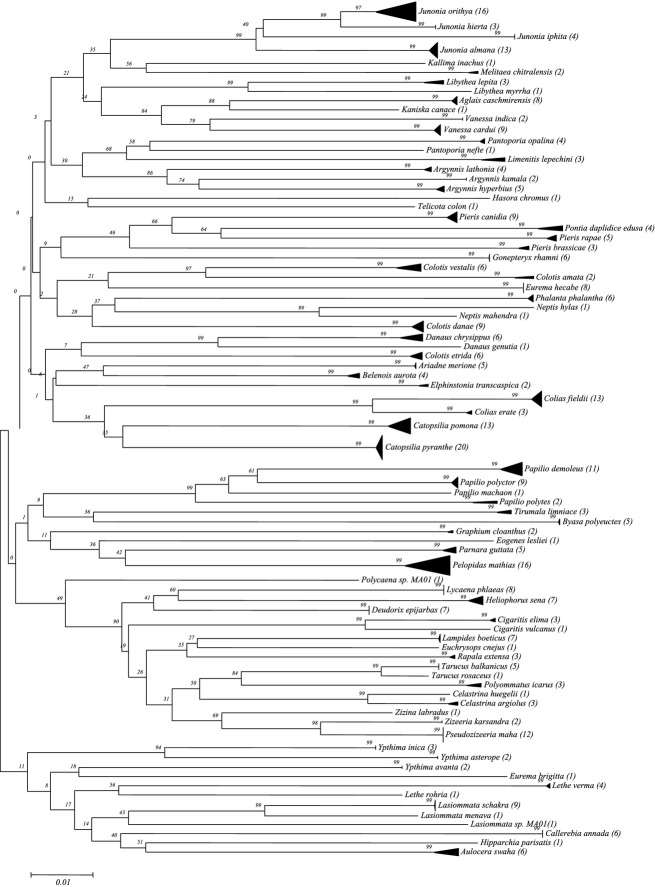
NJ cluster analysis of butterfly species from north-central Pakistan based on the analysis of 374 sequences from 81 species. Bootstrap values (500 replicates) are shown above the branches. The scale bar shows K2P distances. The node for each species with multiple specimens was collapsed to a vertical line or triangle, with the horizontal depth indicating the level of intraspecific divergence. Bracketed numbers next to each species name indicate the number of individuals analysed. Analyses were conducted in mega5.

NJ clustering analysis showed that each of the 81 species formed a monophyletic cluster (Fig. 3). Species with two or more barcode sequences were analysed for species identification using TaxonDNA. When a 3% threshold was employed, 100% of the species were correctly identified using the ‘Best Match or Best Close Match’ criterion. Analysis of the 374 sequence records using TaxonDNA led to the recognition of 78 clusters at a 3% threshold and 80 clusters at a 2% threshold. At the 3% threshold, 75 of the 78 clusters were comprised of a single species, with the largest pairwise intraspecific distance being 2.88%, while 79 of the 80 clusters were a single species at the 2% threshold with the largest pairwise intraspecific distance being 1.67%.

**Fig 4:**
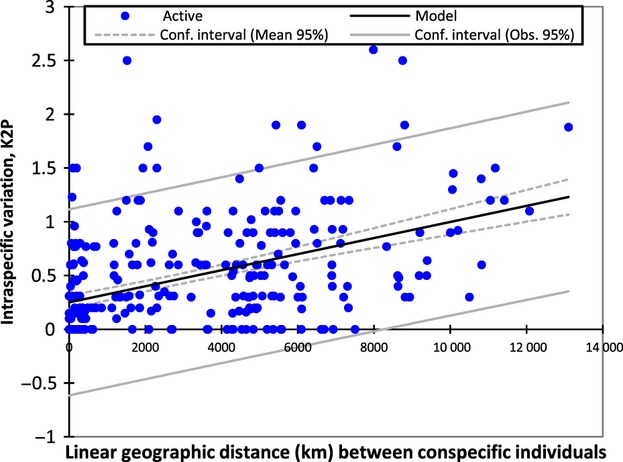
Intraspecific variation (K2P) against geographical extent (km) of butterflies from Pakistan and their conspecifics from other regions (linear regression, *y* = 8E-05*x* + 0.250; *R*^2^ = 0.22).

Genetic divergences increased with taxonomic rank (Table 1; Fig 2) with little overlap between conspecific and congeneric distances. Intraspecific divergences ranged from 0.0 to 1.6% with a mean of 0.2%, while divergences for the species in a genus ranged from 1.7 to 14.3% with a mean of 8.0%. The distances within families ranged from 3.9 to 19.2% with a mean of 13.1%. Fifty-five species were represented by at least one conspecific from another country, but in most cases, there was little increase in intraspecific divergence when they were included in the analysis (Table 2). Seventeen species showed a three-fold or more increase in intraspecific distances (Table 2, bold-faced numbers), but their maximum intraspecific divergence remained <3%, and mean divergence was <1% in all cases except *Colotis amata* (max = 3.20%, mean = 1.17%) (Table 2). The relationship between geographical and genetic distances was quantified by plotting geographical distances against intraspecific variation (K2P). Table 2 provides species-wise Mantel correlation statistics, while Fig. 4 shows the overall trend between geographical distance and intraspecific genetic divergence. Some species showed a strong correlation between the two parameters, as genetic distances increased with geographical distance, but others did not show a significant relationship between the two variables (Table 2). Overall, this analysis showed a weak relationship (*R*^2^ = 0.22; *y* = 8E-05*x* + 0.250) between the geographical extent of a species and its maximum intraspecific divergence (Fig. 4).

**Table 1:** Percentage K2P sequence divergence at the COI barcode region among the 59 butterfly species with >2 specimens, among the 19 genera with two or more species and among the five families with two or more genera

Distance class	*n*	Taxa	Comparisons	Min (%)	Mean (%)	Max (%)
Intraspecific	352	59	1349	0	0.2	1.6
Congeners	233	19	1274	1.7	8.0	14.3
Confamilial	372	5	16 200	3.9	13.1	19.2

**Table 2:** Maximum intraspecific distances for 55 butterfly species with barcode records from Pakistan and other nations

		Maximum intraspecific distance (individuals)			Mantel correlation statistics for geographical vs. genetic distances
No.	Species	Pakistan	Combined	Countries with matches	(α = 0.05)
1	*Aglais caschmirensis*	0.15 (8)	0.2 (12)	Kyrgyzstan, Mongolia, Nepal, Russia, Uzbekistan	*r* = 0.8; *P* = 0.038
2	*Argynnis kamala*	0.0 (2)	0.79 (3)	Nepal	*r* = 1.0; *P* = 0.333
3	*Argynnis hyperbius*	0.46 (5)	0.96 (14)	Australia, Japan, South Korea	*r* = 0.28; *P* = 0.005
4	*Ariadne merione*	0.15 (6)	0.3 (12)	India	*r* = 0.55; *P* = 0.239
5	*Aulocera swaha*	0.92 (6)	0.96 (7)	India	*r* = 0.74; *P* = 0.000
6	*Belenois aurota*	0.46 (4)	0.76 (7)	Kenya	*
7	*Byasa polyeuctes*	0.15 (5)	**0.48** (6)	Taiwan	*r* = 0.71; *P* = 0.064
8	*Catopsilia pomona*	1.39 (13)	1.93 (34)	Australia, China, Papua New Guinea, Thailand, Taiwan	*r* = 0.76; *P* = 0.000
9	*Catopsilia pyranthe*	0.30 (20)	0.36 (13)	Australia, Malaysia	*r* = 0.82; *P* = 0.0001
10	*Celastrina argiolus*	0.31 (3)	**2.2** (76)	Armenia, Canada, Cyprus, Finland, France, Georgia, Germany, Iran, Italy, Kazakhstan, Mexico, Morocco, Romania, Russia, Spain, South Korea, United States	*r* = 0.81; *P* = 0.0001
11	*Colias erate*	0.15 (3)	0.15 (4)	Kyrgyzstan	*r* = −0.27; *P* = 0.708
12	*Colias fieldii*	0.61 (13)	0.64 (16)	China	*r* = 0.93; *P* = 0.0001
13	*Colotis amata*	0.6 (2)	**3.2** (44)	Angola, Iran, Kenya, Madagascar, Namibia, Oman, South Africa, Somalia, Tanzania, Yemen	*r* = 0.27; *P* = 0.042
14	*Colotis danae*	0.64 (9)	1.53 (6)	Iran	*
15	*Colotis etrida*	0.35 (6)	0.35 (8)	India	*
16	*Colotis vestalis*	0.92 (6)	1.4 (21)	Algeria, Ethiopia, Iran, Israel, Oman, Sudan, Yemen	*r* = −0.17; *P* = 0.39
17	*Danaus chrysippus*	1.08 (6)	1.47 (48)	Egypt, India, Italy, Kenya, Madagascar, Morocco, Philippines, Spain, South Africa, Taiwan, Tanzania	*r* = 0.007; *P* = 0.941
18	*Danaus genutia*	– (1)	0.8 (6)	India, Malaysia, Taiwan	*r* = −0.96; *P* = 0.0001
19	*Deudorix epijarbas*	0.0 (7)	0.0 (8)	Taiwan	Genetic distances are ‘zero’
20	*Eurema hecabe*	0.0 (8)	**1.2** (34)	Australia, China, India, Japan, Korea, Malaysia, Papua New Guinea, Sri Lanka, Thailand	*r* = 0.14; *P* = 0.559
21	*Hasora chromus*	– (1)	0.64 (8)	Australia, Papua New Guinea	*r* = −0.52; *P* = 0.001
22	*Hipparchia parisatis*	– (1)	1.22 (4)	Iran	*
23	*Junonia almana*	0.34 (13)	1.02 (14)	India, Malaysia	*r* = 0.77; *P* = 0.008
24	*Junonia hierta*	0.0 (3)	**2.05** (41)	India, Kenya, Madagascar, South Africa, Tanzania	*r* = −0.46; *P* = 0.186
25	*Junonia iphita*	0.0 (4)	**0.92** (8)	India	*r* = 1.0; *P* = 0.333
26	*Junonia orithya*	1.39 (16)	2.0 (19)	Australia, India, Malaysia, Taiwan	*r* = 0.21; *P* = 0.55
27	*Kallima inachus*	– (1)	0.31 (3)	India	*
28	*Kaniska canace*	– (1)	0.8 (8)	Malaysia, South Korea	*r* = −0.97; *P* = 0.0001
29	*Lampides boeticus*	0.16 (7)	**2.63** (126)	Australia, Cyprus, Germany, Egypt, Iran, Israel, Italy, Kenya, Madagascar, Morocco, Papua New Guinea, Portugal, Romania, Spain, Taiwan, Tanzania	*r* = 0.35; *P* = 0.075
30	*Lasiommata menava*	– (1)	1.22 (4)	Iran, Tajikistan	*r* = 0.89; *P* = 0.167
31	*Lasiommata schakra*	0.16 (9)	0.16 (15)	Nepal	*r* = 0.42; *P* = 0.707
32	*Lethe rohria*	– (1)	1.07 (2)	China	*
33	*Lethe verma*	0.31 (4)	**1.12** (6)	China	*r* = 0.93; *P* = 0.039
34	*Libythea lepita*	0.61 (3)	0.92 (8)	South Korea, Taiwan	*r* = 0.13; *P* = 0.789
35	*Limenitis lepechini*	0.77 (3)	0.77 (7)	Uzbekistan	*r* = 0.0; *P* = 0.0001
36	*Lycaena phlaeas*	0.0 (8)	**1.12** (107)	Armenia, Canada, Cyprus, Finland, France, Germany, Iran, Italy, Morocco, Nepal, Norway, Portugal, Romania, Russia, Spain, Tunisia, USA	*r* = 0.7; *P* = 0.0001
37	*Neptis hylas*	– (1)	1.7 (6)	India	*
38	*Papilio demoleus*	0.96 (11)	1.02 (11)	Taiwan	*r* = 0.31; *P* = 0.331
39	*Papilio machaon*	– (1)	2.9 (110)	Canada, Finland, France, Germany, Israel, Italy, Japan, Morocco, Nepal, United States, Russia, Spain, Romania, South Korea	*r* = 0.23; *P* = 0.001
40	*Papilio Polyctor*	0.31 (9)	**1.95** (18)	China	*r* = 0.99; *P* = 0.046
41	*Papilio polytes*	0.8 (2)	1.67 (10)	Malaysia, Thailand	*r* = 0.51; *P* = 0.347
42	*Pelopidas mathias*	1.6 (16)	2.6 (23)	Indonesia, Madagascar, South Africa, UAE	*r* = 0.47; *P* = 0.177
43	*Phalanta phalantha*	0.15 (6)	0.2 (10)	India	*
44	*Pieris brassicae*	0.31 (3)	**1.53** (63)	Armenia, Austria, Finland, France, Germany, Italy, Kyrgyzstan, Morocco, Portugal, Romania, Russia, Spain	*r* = 0.02; *P* = 0.895
45	*Pieris canidia*	0.31 (9)	0.8 (13)	Kyrgyzstan, Uzbekistan	*r* = 0.78; *P* = 0.161
46	*Pieris rapae*	0.30 (5)	0.31 (6)	Nepal, South Korea	*r* = 0.39; *P* = 0.000
47	*Pontia daplidice edusa*	0.77 (4)	1.25 (41)	Armenia, Austria, Finland, Georgia, Germany, Iran, Israel, Italy, Kazakhstan, Romania, Russia, UAE	*r* = 0.04; *P* = 0.689
48	*Pseudozizeeria maha*	0.0 (12)	**0.19** (18)	Japan, South Korea, Taiwan	*r* = 0.97; *P* = 0.068
49	*Tarucus balkanicus*	0.0 (5)	**2.23** (22)	Cyprus, Egypt, Israel, Morocco, Tunisia, Turkey, UAE	*r* = −0.69; *P* = 0.963
50	*Telecota colon*	– (1)	0.77 (5)	Australia	*
51	*Tirumala limniace*	0.62 (3)	**2.09** (11)	India, Kenya, Tanzania	*r* = 0.85; *P* = 0.133
52	*Vanessa cardui*	0.49 (9)	**1.61** (115)	Algeria, Armenia, Australia, Canada, Eritrea, Finland, France, Germany, Israel, India, Italy, Japan, Kazakhstan, Kenya, Morocco, Romania, Russia, South Africa, South Korea, Spain, Taiwan, Tanzania, UAE, USA	*r* = 0.09; *P* = 0.329
53	*Vanessa indica*	0.0 (2)	**0.66** (6)	South Korea, Taiwan	*r* = 0.11; *P* = 0.932
54	*Zizeeria karsandra*	0.0 (2)	**1.53** (14)	Algeria, Australia, Cyprus, Egypt, UAE	*r* = 0.4; *P* = 0.247
55	*Zizina labradus*	– (1)	2.42 (86)	Australia, Kenya, New Zealand, Papua New Guinea, Tanzania	*r* = 0.29; *P* = 0.2

Species from Pakistan with no matches in the databases (*n *=* *26): *Argynnis lathonia*,*Callerebia annada*,*Celastrina huegelii*,*Cigaritis elima*,*Cigaritis vulcanus*,*Elphinstonia transcaspica*,*Eogenes lesliei*,*Euchrysops cnejus*,*Eurema brigitta*,*Gonepteryx rhamni*,*Graphium cloanthus*,*Heliophorus sena*,*Lasiommata sp. MA01*,*Libythea myrrha*,*Melitaea chitralensis*,*Neptis mahendra*,*Pantoporia nefte*,*Pantoporia opalina*,*Parnara guttata*,*Polycaena sp. MA01*,*Polyommatus icarus*,*Rapala extensa*,*Tarucus rosaceus*,*Ypthima avanta*,*Ypthima sakra*,*Ypthima inica*

The number of individuals of a species included in the analysis is indicated in brackets. A double dash indicates that a given species was presented by only one specimen, and thus, maximum intraspecific divergence is not presented, while bold highlighting is used to indicate those species that exhibit a three-fold or greater increase in intraspecific variation when records outside of Pakistan were included.

* Insufficient data to run the Mantel test.

## Discussion

### Identification success for the butterflies of Pakistan

This study has begun the construction of a DNA barcode reference library for the butterflies of Pakistan. Cluster analysis revealed that all 81 species examined in the study formed a monophyletic cluster which corresponded perfectly with the taxa recognized on morphological criteria. Although three species pairs showed limited divergence (<3%), maximum intraspecific divergence was always lower than the NN distance, enabling the separation of all species. Even the most closely related (1.70%) species pair, *Tarucus balkanicus* and *T. rosaceus*, was separated with strong bootstrap support in the NJ tree. Our results confirm the usefulness of DNA barcoding in identifying the butterflies of Pakistan, but the sample size was low for some species and 75% of the fauna awaits analysis.

When sequences for butterfly species from Central Asia (Lukhtanov *et al*. 2009) were included, eight species pairs formed paraphyletic clusters. Among these pairs, the NN distance between *Aglais caschmirensis* (from Pakistan) and *A. nixa* (from Uzbekistan) was 0.2%, while that between *A. caschmirensis* and *A. urticae* (from Kazakhstan) was 1.4%. Although NN distances for these sister species pairs were small, barcode-based identifications were possible as reported by Tavares & Baker (2008) in their study on sister species of birds.

‘Barcode Gap Analysis’ showed that NN distance for all the species was higher than the maximum intraspecific distance. The Barcode Index Number (BIN) system (Ratnasingham & Hebert 2013) provided further evidence of the genetic distinctiveness of the species as it assigned the 81 species to 80 BINs with only *T. balkanicus* and *T. rosaceus* sharing a BIN. When identity analysis was performed using Best Match/Best Close Match at a 3% threshold, all the species were correctly identified. Other studies have generally reported similar results (Janzen *et al*. 2005; Lukhtanov *et al*. 2009; Gaikwad *et al*. 2012) with a few exceptions. For example, Gaikwad *et al*. (2012) found that intraspecific divergence was higher (7.8%) in the butterfly *Lethe europa* than the distance to its NN (7.4%). Such cases can, of course, arise through a failure to discriminate sibling taxa. Bortolus (2008) has emphasized the importance of detailed taxonomic study in cases where DNA barcode results are discordant with taxonomic assignments. Costa *et al*. (2012) have reinforced this conclusion, noting the need for a ranking system to register the certainty of identifications for specimens used to develop reference barcode libraries. These suggestions reinforce the importance of an integrative approach to species delimitation by considering morphological, genetic, ecological and geographical information, rather than considering taxonomic identifications as facts against which to ‘test’ DNA barcoding (e.g. Smith *et al*. 2008). Nevertheless, focusing on one region of the genome is useful to the community for generating a comparable set of sequences across a large number of diverse taxa and geographical regions.

### Genetic divergence patterns with increasing geographical distance: a regional Asian perspective

The within-species divergence values for most species in the study were under the 2%. In most cases, the addition of conspecific sequences from other countries increased the intraspecific distance, but the relationship between geographical distance and the level of intraspecific divergence was not strong. In a few cases, substantial intraspecific distances were observed between specimens from the same region. For example, *Pelopidas mathias* collected from sites in Pakistan <250 km apart showed 1.54% divergence. On the other hand, *Deudorix epijarbas* from Pakistan and Taiwan (4832 km) lacked barcode divergence. Other species showed regional variation that was not linked to distance. For example, specimens of *Lampides boeticus* from Pakistan and Queensland Australia were just 0.4% divergent, but specimens from Papua New Guinea were 1.9% divergent. These results reinforce previous conclusions that geographical distance is often associated with an increased genetic divergence, but that the increase is too small to impede the identification of species (Lukhtanov *et al*. 2009; Bergsten *et al*. 2012; Gaikwad *et al*. 2012).

### Diversity hotspots and endemism in Asia underscores the need for regional barcode libraries

Although Pakistan and neighbouring Central Asia are only 700 km apart, prior studies have indicated that there is little overlap in their butterfly faunas. In fact, just 42 species (14%) are shared among the 320 butterfly species from Pakistan (Roberts 2001) and the 353 species from Central Asia (Lukhtanov *et al*. 2009). Their distinctive faunas undoubtedly reflect the effectiveness of the Pamir mountain chain, which rises to more than 5000 m, as a dispersal barrier. This limited overlap suggests the presence of multiple regions of endemism in this segment of Asia, mirroring a pattern of low overlap between the biodiversity hotspots in the Western Ghats (India) and Sri Lanka (Bossuyt *et al*. 2004). Although India and Sri Lanka are on the same continental shelf, and the strait separating them does not exceed 70 m in depth, limited biotic interchanges have left the two areas with an unexpectedly large number of endemics. This fact highlights the need to expand barcode coverage for all animal groups from the various subregions in southern Asia. Certainly, barcode reference libraries based on species from other nations will only permit the identification of a fraction of Pakistan's biodiversity.

## Notes

M.A. designed and performed experiment, analyzed data and wrote the paper. S.A. and A.M.K. collected and identified butterflies. S.J.A. provided help with analysis and writing the paper. P.D.N.H. designed the methods, contributed reagents and wrote the paper.

## Data Accessibility

Specimen data, images and DNA sequences: BOLD project MABUT (Barcoding Butterflies of Pakistan).

DNA sequences: BOLD IDs and GenBank accession nos for each butterfly specimen listed in Table S1 (Supporting information).

Sequence alignments: Supporting information.

## Supporting information

Appendix S1 ClustalW alignment of barcode sequences of 81 butterfly species from Pakistan.

Table S1 BOLD IDs and GenBank accessions of butterfly specimens included in the study.
